# Rare cause of bilateral groin swelling: Round ligament varicosities

**DOI:** 10.12669/pjms.314.7954

**Published:** 2015

**Authors:** Erdogan Bulbul, Mine Islimye Taskin, Bahar Yanik, Gulen Demirpolat, Ertan Adali, Murat Basbug

**Affiliations:** 1 Erdogan Bulbul, Department of Radiology, Balikesir University Faculty of Medicine, Balikesir, Turkey; 2 Mine Islimye Taskin, Department of Obstetrics & Gynecology, Balikesir University Faculty of Medicine, Balikesir, Turkey; 3 Bahar Yanik, Department of Radiology, Balikesir University Faculty of Medicine, Balikesir, Turkey; 4 Gulen Demirpolat, Department of Radiology, Balikesir University Faculty of Medicine, Balikesir, Turkey; 5 Ertan Adali, Department of Obstetrics & Gynecology, Balikesir University Faculty of Medicine, Balikesir, Turkey; 6 Murat Basbug, Department of Surgery, Balikesir University Faculty of Medicine, Balikesir, Turkey

**Keywords:** Pregnancy, Round ligament, Ultrasonography, Varicosity

## Abstract

Round ligament varicosity (RLV) is rare and almost all cases are pregnant women. RLV appears as a unilateral or bilateral groin swelling. Pain and tenderness may present. Clinical evaluation is inadequate for exact diagnosis because inguinal hernia has similar findings. Ultrasonography (US) is essential when a groin swelling is detected in a pregnant woman. We present gray scale US and colour Doppler US findings of a 32-week pregnant woman with bilateral RLVs at the inguinal canal, parauterine area and in the myometrium.

## INTRODUCTION

Groin swelling becomming apparent during the pregnancy may cause a clinical confusion. Round ligament varicosities (RLVs) and inguinal hernia are common causes of groin swelling during pregnancy. Symptoms and clinical findings are similar so clinical evaluation is inadequate in differential diagnosis.^1^,^2^ It is important to establish the definite diagnosis for management. Gray scale ultrasonography (US) and colour Doppler US are most appropriate modalities for the exact diagnosis.^2^ Herein, we present a 32-week pregnant woman with bilateral RLVs extends to myometrium who presented with bilateral groin swelling.

## CASE REPORT

**Fig. 1:**
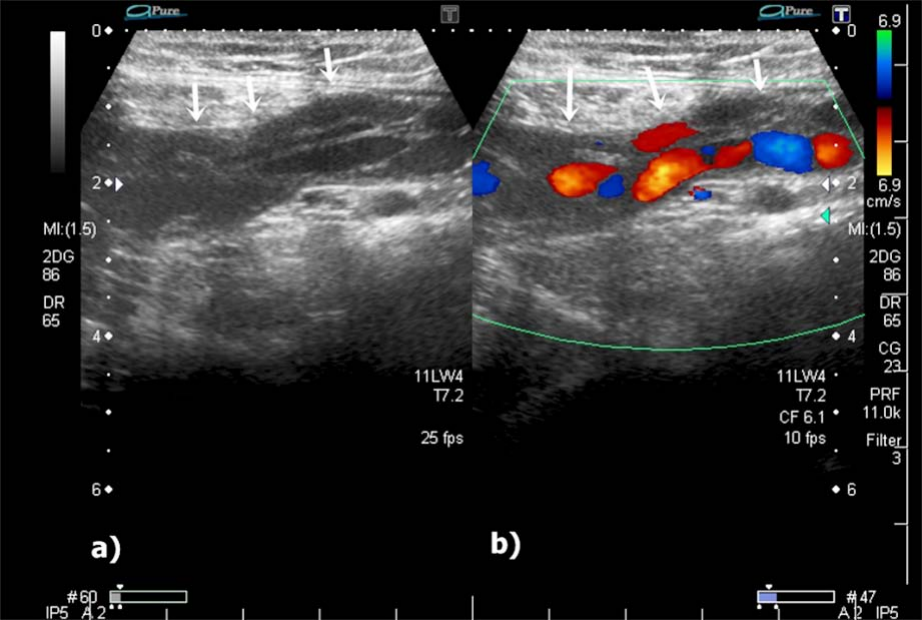
Gray scale and colour Doppler ultrasonography (US) reveals dilated, tortuous channels (white arrows) at the left inguinal region (a, b).

**Fig. 2:**
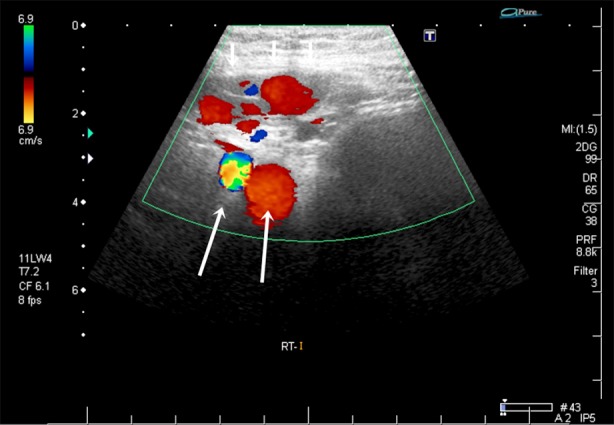
The axial image shows dilated veins (white arrows) at the right inguinal canal. Long arrows show the right iliac artery and vein.

**Fig. 3:**
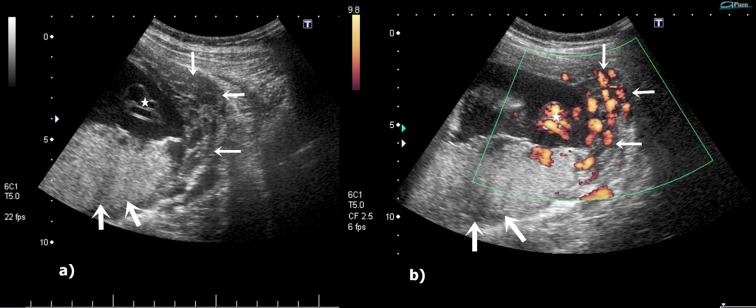
Gray scale and power Doppler US demonstrates varicosities extending into the myometrium (white arrows). Placenta is shown with thick arrows and umbilical vessels with star respectively (a, b).

A 32- year old pregnant woman, parity 1, presented with bilateral painless groin swelling. Her first pregnancy was uneventful. Groin swelling firstly appeared on the left side at 16 week of second pregnancy followed by the other side. The masses gradually increased and patient was consulted by a surgeon at the 32rd gestational week. Bilateral reducible soft masses with enlargement by cough impulse were detected at physical examination. Patient was referred to radiology department for ultrasound evaluation. The gray scale US and colour Doppler US of the inguinal canal was performed with a Siemens-Acuson S2000 scanner (Siemens Healthcare, Erlangen, Germany) and a 4-9 MHz multifrequency linear-array transducer. Ultrasound revealed bilateral inguinal anechoic, compressible, tubular, tortuous channels. Venous flow detected within the channels by colour Doppler US and spectral analysis. The lesions showed continuity through the internal inguinal ring and extended to parauterine space and myometrium (Fig. 1, 2 and 3). Tubular channels were also seen through the labium major. The lesions became prominent with Valsalva manoeuvre and erect position. Based on the ultrasound findings RLVs diagnosed. The patient was closely followed-up, and had an elective caesarean section. Groin swelling regressed within four weeks without any complications.

## DISCUSSION

Round ligaments attaches uterus on each sides, extends laterally, passes through the inguinal canal and terminates at the labia major. Round ligaments contains arteries, veins, lymphatics and nerves, enables the anteverting position of the uterus.^3^ Dilatation of surrounding veins is called as RLVs. RLVs may extend from myometrium, parauterine area to labium major including inguinal canal.^1^ Vulvar and lower extremity varicosities may be seen with RLVs.^2^,^4^

Limited number of cases about RLVs reported in the literature.^1^,^5^ RLV incidence is not exactly known but McKenna et al. observed 5 cases in 3186 deliveries.^6^ RLV is usually unilateral but one third of the cases in literature are bilateral.^5^ In our case RLV firstly appeared at the left side and within a few weeks right sided swelling occurred.

In a recently published article authors emphasised that groin swelling firstly appeared during pregnancy is due to RLVs more than inguinal hernias.^7^ Pregnancy causes a predisposition for RLV development yet nearly all of the known cases are pregnant women. Only one non-pregnant case was reported.^5^ Physiopathology can be explained by progesterone-mediated smooth muscle relaxation, increased cardiac output and intravascular fluid volume, increased venous return from lower extremities and compression of veins due to growing uterus.^2^

RLVs present as a groin swelling at the second or third trimester of pregnancy.^4^ Tenderness and pain may be concomitantly. Physical examination reveals partially or considerably reducible mass. Mass may enlarge with Valsalva manoeuvre and on erect position. Clinical findings are inadequate in differentiation of RLV and inguinal hernia therefore ultrasound evaluation is necessary in a pregnant woman presenting with a new–developing groin swelling.^8^

US and colour Doppler US are available in differentiation of uncomplicated RLV and inguinal hernia. US characteristics of RLV are multiple dilated veins at the inguinal canal, absence of lymph nodes or bowel in the inguinal canal, detecting veins draining to inferior epigastric artery.^6^ Another typical finding is “bag of worms” appearance of the smaller varices.^4^ Colour Doppler US confirms the venous flow and augmentation of venous flow with Valsalva manoeuvre. RLVs may extend into the pelvis. Dilated veins can be detected in the myometrium and adjacent to uterus with US and colour Doppler US.^1^ In our case this rare finding was also detected. Peristaltic bowel segment or mesenteric fat containing mass within the inguinal canal are the US findings of inguinal hernia.^9^ In our case intestinal segments or mesenteric fat was not detected in the inguinal regions.

Both thrombosed RLV and incarcerated hernias are indiscernible by clinical findings. Pain, tenderness and uncompressible mass are mutual clinical findings. It is important to differentiate these pathologies to prevent unnecessary operations and US is also helpful in complicated cases.^10^ Uncompressible, clot containing tubular channels without flow should be considered as thrombosis of RLV.^5^ US findings of incarcerated hernia are bowel wall thickening and fluid in the hernia sac, dilated bowel-loops in abdominal cavity.^11^

Other pathologies in differential diagnosis of RLV beyond the inguinal hernia are lymphadenopathy, endometrioma, abscess, hematoma, cyst, lipoma and lymphangioma. US and colour Doppler US are also helpful in characterisation of these pathologies.^12^

The exact diagnosis is important in management. Both RLV and inguinal hernia are closely followed-up as “wait and see” principle. Uncomplicated RLV is treated conservatively and expected to regress within a few months after delivery.^5^,^6^,^8^ Uncomplicated inguinal hernias should be operated after delivery.^7^ In our case complication did not occur during pregnancy and postpartum period. RLV regressed within four weeks.

In conclusion RLVs are rare cause of groin swelling and almost always seen in pregnant women. RLVs may not be limited to inguinal region and extend to parauterine and intrauterine regions. The radiologists and clinicians should keep in mind this diagnosis in pregnant women with groin swelling. US and colour Doppler US findings are not only useful in diagnosis but also helpful in determining the extension of RLVs.
